# Is the association of QTc with atrial fibrillation and stroke in cohort studies a matter of time?

**DOI:** 10.1136/openhrt-2022-002080

**Published:** 2022-09-28

**Authors:** Navid Radnahad, Hanne Ehrlinder, Karin Leander, Johan Engdahl, Håkan Wallén, Bruna Gigante

**Affiliations:** 1 Department of Clinical Sciences, Division of Cardiovascular Medicine, Danderyd University Hospital, Karolinska Institutet, Stockholm, Sweden; 2 Unit of Cardiovascular and Nutritional Epidemiology, Institute of Environmental Medicine, Karolinska Institutet, Stockholm, Sweden; 3 Division of Cardiovascular Medicine, Department of Medicine, Karolinska Institutet, Stockholm, Sweden

**Keywords:** atrial fibrillation, risk factors, stroke

## Abstract

**Objectives:**

To investigate the association of the heart rate-corrected QT interval (QTc) with the risk of atrial fibrillation (AF) and ischaemic stroke.

**Methods:**

We estimated the risk of AF and ischaemic stroke associated with QTc duration (ms) by Cox regression in study participants from the cohort of 60-year-old men and women from Stockholm (60YO) (n=4232). Univariate and multivariate adjusted risk estimates were expressed as HR and 95% CI. Main results were validated in elderly patients with AF, included in the Carebbean-e study, where an ECG in sinus rhythm (SR) (ECG-SR) recorded before the ECG diagnostic for (ECG-AF) was available (n=803). We estimated the correlation between the time interval (years) between the ECG-SR and ECG-AF with the QTc duration, by the Spearman correlation coefficient (rho).

**Results:**

In the 60YO, the highest QTc duration quartile (>427 ms) associated with the AF risk (n=435) with a multivariable adjusted HR of 1.68 and 95% CI (1.26 to 2.24). No association was observed with ischaemic stroke. In the Carebbean-e study, no significant association was observed between the QTc duration measured on the ECG-SR and risk of ischaemic stroke during follow-up. QTc duration showed an inverse correlation (rho: −0.26, p<0.0001) with the time interval intercurred between ECG-SR and ECG-AF.

**Conclusions:**

The association of QTc duration with AF risk might depend on the time interval between the QTc measurement and the clinical diagnosis of AF. No association was observed between QTc duration and ischaemic stroke.

**What is already known on this topic**

- Heart rate-corrected QT interval (QTc) duration has been associated with the risk of atrial fibrillation (AF) and ischaemic stroke in observational studies.

**What this study adds**

- We report that the QTc interval might be considered an AF intermediate phenotype, being longer in close proximity of the AF diagnosis.

**How this study might affect research, practice or policy**

- As an ECG is recorded in each patient with suspected AF, QTc can be readily measured. Presence of a progressive longer QTc duration may reinforce the indication to AF screening as this may be a sign of impedent or paroxysmal AF.

## Introduction

Atrial fibrillation (AF) increases the risk of ischaemic stroke by a factor of five.^1^ As no cure exists, prevention and identification of risk indicators for AF and ischaemic stroke is of utmost importance for the single individual and society.

ECG is a universally used method to diagnose AF. As almost every patient undergoes an ECG, the validation of ECG parameters able to improve AF and ischaemic stroke risk stratification may help the clinician to identify patients who may benefit from antithrombotic treatment to prevent ischaemic stroke, above and beyond the currently used risk scores.

The length of the heart rate-corrected QT interval (QTc) has been associated with the risk of atrial arrhythmias.^2^ For instance, a J-shaped curve has been proposed to explain the association between QTc duration and AF risk. Both a long and a short QTc have been associated with an increased AF risk in family-based genetic studies^3 4^ and in open large populations.^5 6^ A prolonged QTc has also been associated with an increased risk of ischaemic stroke, after adjustments for AF and other known cardiovascular risk factors.^7 8^ However, the association of QTc with the risk of ischaemic stroke in the presence and absence of known AF is still debated.^7 8^

To this end, we have investigated the association of QTc duration with the risk of AF and with the risk of ischaemic stroke in a cohort of 60 years old men and women from Stockholm (60YO), free of cardiovascular event and AF at inclusion. We have validated our findings in patients with newly diagnosed AF by ECG included in the Carebbean-elderly (Carebbean-e), a cohort of hospitalised elderly patients with AF^9^ using the QTc measured on an ECG in sinus rhythm (SR) (ECG-SR) recorded before the ECG diagnostic for AF (ECG-AF). In this cohort, we have also estimated the correlation between QTc duration and the time elapsed between the two ECGs, ECG-SR and ECG-AF.

## Methods

### Study population

The 60YO has been previously described in detail.^10–12^ Briefly, every third man and woman living in Stockholm County reaching the age of 60 between 1 July 1997 and 30 June 1998 was invited to participate in a cardiovascular health screening. The participants (n=4232) completed a comprehensive validated questionnaire which collected information regarding their lifestyle habits, medication and prior diseases. They underwent a physical examination including a 12-lead resting ECG, and blood samples were taken and stored at −80℃ until analysis.

For the present analysis, study participants with prevalent coronary heart disease, ischaemic stroke and/or AF were excluded (n=369). The National Patient Register (NPR) was used to ascertain prevalent diseases using the following International Classification of Diseases 10th revision (ICD-10) codes: I48.- (AF) which include atrial flutter (AFL), I21–I25.- (MI) and I63.- (ischaemic stroke) and/or if it was self-reported and/or, for AF, in the presence of a diagnostic ECG. In addition, study participants without a readable ECG or bearing a pacemaker (n=277) and/or without complete information from the questionnaire (n=122) were excluded, leaving 3464 study participants eligible for the analysis. Online supplemental figure I, panel A summarises the exclusion criteria applied in the 60YO.

10.1136/openhrt-2022-002080.supp1 Supplementary data

### Outcome ascertainment

Incident AF or AFL and fatal/non-fatal ischaemic stroke diagnoses were collected from the NPR and Swedish Cause of Death Register using ICD-10 codes I48.- and I63.-. until 31 December 2017. No consideration was taken whether AF or AFL was the main or bi-diagnosis, or if the AF or AFL diagnosis preceded or followed the ischaemic stroke diagnosis.

### ECG parameters

ECG parameters measured in the 60YO were heart rate (bpm), PQ, QRS and QTc duration expressed in milliseconds (ms). They were automatically measured from 12-lead ECG recorded at inclusion. Left ventricular hypertrophy (LVH) was defined by ECG using two established criteria, the Minnesota Code and the Cornell voltage-duration product, as previously reported.^13^

### Validation cohort: the Carebbean-e

The Carebbean-e is a prospective cohort designed to investigate the clinical determinants of thromboembolism and bleeding in AF/AFL elderly (≥75 years) patients.^9^ Briefly, 2943 consecutive patients discharged from Danderyds University Hospital from 1 November 2010 to 31 December 2017 with AF/AFL as main diagnosis were included in the study. ECG at admission showing AF or AFL was stored in medical records.

In the present study, we only included patients with newly diagnosed AF or AFL at inclusion, who had not suffered an ischaemic stroke (ascertained from the NPR using congruent diagnosis codes or if self-reported at admission) (n=1146). Among those, we included only patients with a readable ECG registered before inclusion (n=813). The most recent available ECG-SR registered in the medical records available at our hospital before the ECG-AF registered at admission was retrospectively revised and used to measure the QTc duration. The time interval elapsed between the two ECGs (ECG-AF and ECG-SR) was calculated and expressed in years (years). Incident ischaemic stroke cases were collected from the NPR and National Cause of Death Register in Sweden using ICD-10 codes I63.-. until 31 December 2019. Online supplemental figure I, panel B summarises the exclusion criteria applied in the Carebbean-e.

### Patient and public involvement

It was not possible to involve patients or the public in the design, or conduct, or reporting, or dissemination plans of our research as this was not a common procedure when both the 60YO and the Carebbean-e studies were designed and analyses conducted.

### Statistical analysis

Continuous variables are expressed as median with IQR and categorical values as numbers and percentage. In the 60YO, the risk for AF and ischaemic stroke associated with QTc duration (ms) was estimated by Cox proportional hazards regression model and expressed as HR with 95% CI. QTc was introduced in the model as a continuous variable and after categorisation in quartiles. Quartiles (Q) boundaries were from the lowest quartile Q1<397; Q2≥397, <411; Q3≥411, <427 and Q4≥427. Prolonged QTc interval was defined as a QTc≥450 ms in men and ≥460 ms in women.^14^ We ran three regression models to analyse the association of QTc duration with (1) the risk of incident AF, (2) the risk of incident ischaemic stroke and (3) the risk of ischaemic stroke in patients with AF. In the first regression model, we considered only incident AF (ie, without a diagnosis of incident ischaemic stroke during follow-up) as outcome and data were censored for incident AF, death or end of the follow-up. In the second model, we considered incident ischaemic stroke (ie, without an AF diagnosis during follow-up) as outcome and data were censored for incident ischaemic stroke, death or end of the follow-up. In the third model, we considered as outcome ischaemic stroke and AF. Data were censored for AF and ischaemic stroke, death and end of follow-up which ever came first. Crude risk estimates were adjusted for sex, hypertension (defined as current treatment for hypertension and/or blood pressure levels at the inclusion ≥140/90 mm Hg and/or self-reported), LVH and QRS interval length in model 1 and additionally adjusted by diabetes (defined as current treatment with hypoglycaemic drugs and/or insulin and/or serum glucose levels at the inclusion ≥7.0 mmol/L and/or self-reported), smoking (categorised as current smoker, former smoker if the study participant had stopped smoking at least 2 years before inclusion and non-smoker) and hyperlipidaemia (defined as current treatment cholesterol lowering drugs and/ or fasting total cholesterol >5.0 mmol/L and/or self-reported), body mass index (BMI) and alcohol overconsumption (g/day) estimated by the daily alcohol consumption and defined as >20 in women and >30 in men,^15^ in model 2.

In the Carebbean-e, the association of the QTc duration with the risk of ischaemic stroke was estimated by Cox proportional hazards regression model using as exposure the QTc measured on the ECG-SR as a continuous variable and categorised into quartiles. QTc (ms) boundaries were from the lowest quartile Q1<414; Q2≥414, <433; Q3≥433, <453; Q4≥453. Crude risk estimates were adjusted for the components of the CHA2DS2-VASc score (age, sex, hypertension (defined as current treatment for hypertension and/or blood pressure levels at the discharge ≥140/90 mm Hg and/or self-reported), heart failure (HF) (left ventricular ejection fraction ≤40 confirmed with echocardiography, and/or presentation with HF symptoms at admission and/or previous admission due to HF), diabetes (defined as treatment with hypoglycaemic drugs and/or insulin and/or self-reported) and previous myocardial infarction and/or known atherosclerotic peripheral diseases (defined as current treatment and/or reported in the medical records) in model 1 and additionally adjusted by the time interval between the two ECGs and QRS length, model 2.

We estimated the correlation between the QTc duration and the time interval (years) between the ECG-AF and ECG-SR. The time interval (years) between the two ECGs was divided in quartiles calculated in the entire study population Q4>5.07; Q3≤5.07 to >2.16; Q2≤2.16 to >0.69; Q1≤0.69. Differences across quartiles were estimated by analysis of variance. The correlation between time from ECG to AF/AFL diagnosis and the QTc duration was estimated by the Spearman correlation coefficient (rho). All statistical analyses were performed using STATA V.15 (College Station, Texas, USA).

### Ethics

The 60YO and the Carebbean-e were designed and conducted in accordance with the Helsinki Declaration.

## Results

Baseline characteristics of study participants from 60YO are reported in table 1 according to the QTc quartiles. Study participants in the highest QTc quartile had a higher BMI and a higher prevalence of hypertension, diabetes and LVH.

**Table 1:** Clinical characteristics of the 60YO study participants included in the present study based on QTc duration categorised in quartiles.

QTc duration				
	Q1<397 ms (n=831)	Q2≥397 to <411 ms (n=898)	Q3≥411 to <427 ms (n=868)	Q4≥427 ms (n=867)
Male/female	477/354	405/493	363/505	360/507
Anthropometric measures				
BMI (kg/m^2^)	26 (24–28)	26 (24–29)	26 (24–29)	27 (24–30)
Systolic blood pressure (mm Hg)	133 (121–147)	134 (120–148)	137 (122–151)	141 (125–157)
Diastolic blood pressure (mm Hg)	82 (76–89)	82 (76–90)	83 (76–91)	85 (78–93)
Risk factors, n (%)				
Smoking, current; former	118 (23); 319 (39)	198 (22); 338 (38)	168 (20); 325 (38)	173 (20); 322 (38)
Hypertension	83 (10)	111 (12)	134 (15)	206 (24)
Diabetes	27 (3)	19 (2)	19 (2)	35 (4)
Hyperlipidaemia	27 (3)	37(4)	31(4)	31 (3)
Alcohol overconsumption, yes; no	701;130	766;132	732;135	754;112
ECG parameters				
Heart rate, bpm	67 (61–73)	65 (58–72)	65 (58–72)	62 (56–70)
PQ, ms	166 (150–182)	163 (148–178)	166 (150–178)	166 (150–182)
QRS, ms	86 (82–92)	88 (82–94)	88 (82–96)	90 (84–100)
QTc, ms	388 (382–393)	404 (400–407)	417 (414–422)	439 (432–453)
LVH, n (%)	37 (5)	48 (5)	68 (8)	103 (12)

Continuous data are reported as median and IQR.

Missing data: smoking, n=45; systolic and diastolic blood pressure, n=2; alcohol consumption, n=2; LVH, n=93.

BMI, body mass index; bpm, beats per minute; LVH, left ventricular hypertrophy; ms, milliseconds; QTc, heart rate-corrected QT interval; 60YO, 60-year-old men and women from Stockholm.

During a mean follow-up time of 19.4 years, 147 incident ischaemic stroke, 435 incident AF and 55 cases with both incident AF and ischaemic stroke were recorded.

### QTc interval and risk of AF and ischaemic stroke in the 60YO

We analysed the association of QTc duration with incident AF, incident ischaemic stroke and incident AF and ischaemic stroke. AF risk estimates increased across QTc duration quartiles as shown in table 2. In the fully adjusted model, the highest QTc quartile was associated with an increased risk of incident AF with an HR of 1.66 and 95% CI (1.22 to 2.24). Consistently, as shown in online supplementary table I, every millisecond increase in the QTc duration was associated with the risk of future AF.

**Table 2:** Association of QTc interval categorised in quartiles with the risk of incident AF, incident ischaemic stroke and incident ischaemic stroke and AF in the 60YO

	Non cases/cases	Q2	Q3	Q4
Atrial fibrillation HR (95% CI)				
Crude	3029/435	1.06 (0.79 to 1.42)	1.28 (0.96 to 1.70)	1.65 (1.26 to 2.16)
Model 1	2946/425	1.18 (0.87 to 1.59)	1.34 (1.00 to 1.80)	1.70 (1.28 to 2.27)
Model 2	2907/421	1.13 (0.84 to 1.53)	1.21 (0.90 to 1.63)	1.66 (1.24 to 2.22)
Ischaemic stroke HR (95% CI)				
Crude	3317/147	0.84 (0.52 to 1.36)	0.96 (0.60 to 1.54)	1.23 (0.79 to 1.92)
Model 1	3228/143	0.85 (0.52 to 1.39)	1.02 (0.63 to 1.64)	1.20 (0.75 to 1.91)
Model 2	3185/143	0.86 (0.53 to 1.41)	0.99 (0.61 to 1.60)	1.24 (0.77 to 1.97)
Atrial fibrillation and ischaemic stroke HR (95% CI)				
Crude	3409/55	1.14 (0.47 to 2.75)	1.84 (0.82 to 4.13)	1.99 (0.89 to 4.44)
Model 1	3318/53	1.38 (0.55 to 3.46)	2.07 (0.87 to 4.92)	2.23 (0.93 to 5.32)
Model 2	3276/52	1.26 (0.49 to 3.22)	2.18 (0.92 to 5.18)	2.37 (0.99 to 5.68)

QTc quartiles (Q) boundaries (ms): Q1<397; Q2≥397, <411; Q3≥411, <427 and Q4≥427. Q1 is the reference group in all analyses.

Model 1: adjusted for sex, hypertension, left ventricular hypertrophy and QRS length.

Model 2: model 1+diabetes, smoking, hyperlipidaemia, BMI and alcohol overconsumption consumption.

Missing values as reported in table 1.

AF, atrial fibrillation; BMI, body mass index; QTc, heart rate-corrected QT interval; 60YO, 60-year-old men and women from Stockholm.

No significant association was observed between QTc duration and the risk of ischaemic stroke in study participants without a diagnosis of incident AF when the QTc duration was categorised in quartiles (table 2) or introduced in the model as a continuous variable (online supplemental table I). The highest QTc quartile showed a borderline association with the risk of AF and ischaemic stroke in the fully adjusted model with an HR of 2.37 and 95% CI (0.99 to 5.68) (table 2) and a nominally statistical significant association when in the model as introduced as a continuous variable (online supplemental table I). A prolonged QTc interval was measured in 276 study participants and was not associated with the risk of ischaemic stroke with HR of 1.06 and 95% CI (0.59 to 1.89) or AF and ischaemic stroke with an HR of 1.06 (0.59 to 1.89).

### Validation in the Carebbean-e study

We investigated the association of QTc duration with risk of AF and ischaemic stroke in the Carebbean-e, a population of elderly patients where AF was verified by ECG. The QTc was measured on the ECG-SR. Online supplemental table II summarises the clinical characteristics of the Carebbean-e patients included in the present study.

We measured the QTc duration on the most recent ECG-SR recorded before the first ECG-AF which was the index event for inclusion in the Carebbean-e study. The ECG-SR were recorded between 24 years and 2 days (median time 2.16 years) before the ECG-AF. During a mean follow-up time of 3.3 years (from the AF diagnosis), 86 patients suffered from ischaemic stroke. No difference in QTc duration (ms) on the ECG-SR was observed between patients suffering from ischaemic stroke and those who remained stroke-free during follow-up after the AF diagnosis 434.5 (413 to 458) versus 433 (414 to 453), p=0.57.

As shown in online supplemental table III, the highest QTc quartile was associated with higher risk estimates for ischaemic stroke, but no statistical significance was attained. Adjustment for the components of the CHA2DS2-VASc score (model 1) and for the time interval between ECG-SR and ECG-AF marginally affected the risk estimates. Similarly, the risk estimate (HR) for ischaemic stroke in the group with prolonged QTc (n=185) as compared with the referent group in the fully adjusted model was 1.24 and 95% CI (0.75 to 2.06).

### Time between ECG recorded in SR and ECG diagnostic for AF and QTc duration

**Figure 1:**
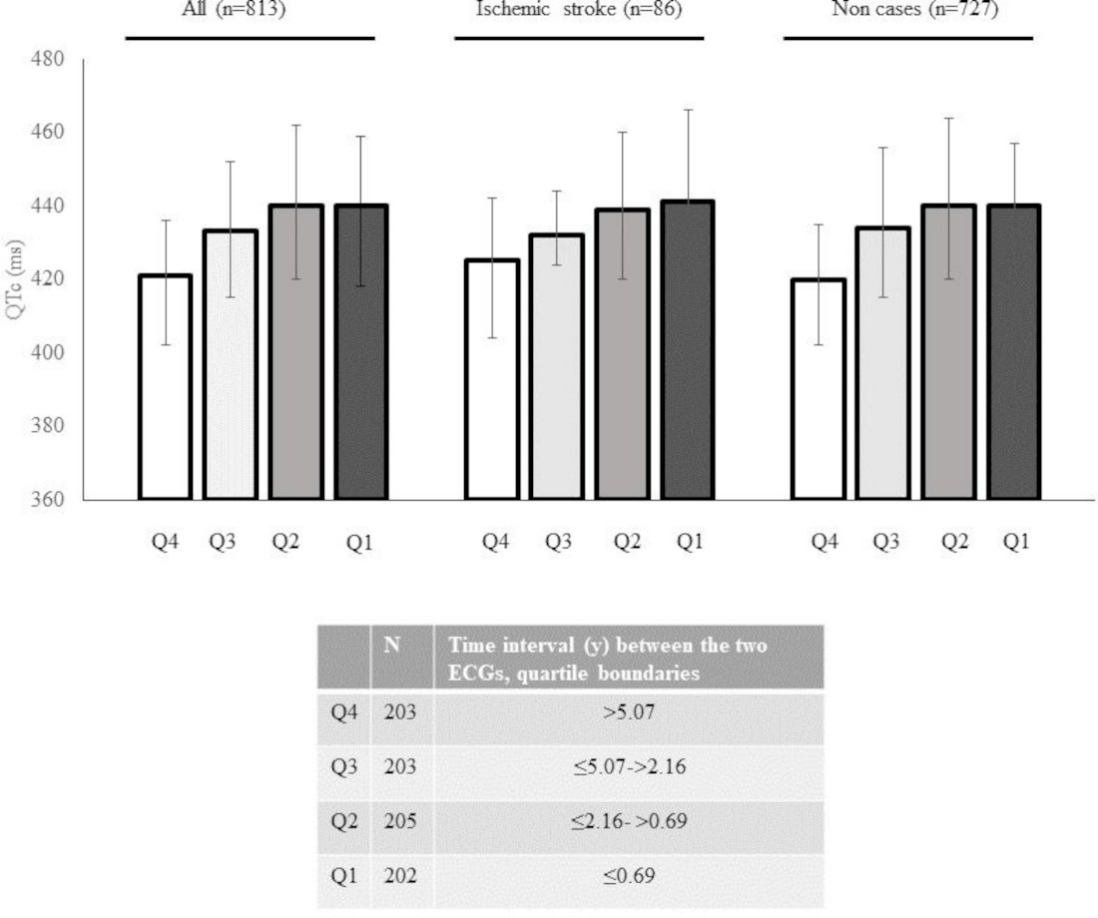
Bar graph showing the QTc duration measured at different time before the AF diagnosis in the Carebbean-e. QTc duration is summarised for all the study participants (n=813), for the group of patients with incident ischaemic stroke at follow-up (n=86) and for those who remained stroke-free (n=727). Time interval (years) between the two ECGs (ECG-SR-ECG-AF) is categorised in quartiles. Quartile boundaries are : Q4>5.07, Q3≤5.07 to >2.16, Q2≤2.16 to >0.69, Q1≤0.69. AF, atrial fibrillation; QTc, heart rate-corrected QT interval.

As shown in figure 1 and summarised in online supplemental table IV, the longest QTc duration (ms) was observed in close proximity to the time of AF diagnosis in the Carebbean-e study. When the time elapse between ECG-SR and ECG-AF was divided in quartiles, QTc duration (ms) was longest 440 (418 to 459) on the ECGs recorded less than 1 year (Q1) before the ECG-AF and shortest when recorded more than 5 years (Q4) before the ECG-AF, 420 (402 to 436), (p<0.0001). The time at which the ECG-SR was recorded inversely correlated with the QTc duration (rho: −0.26, p<0.00001), the shortest the time interval between the two ECGs, the longest the QTc interval. No differences were observed when the patients were divided in two groups, those who suffered from ischaemic stroke during follow-up and those who remained stroke-free (figure 1 and online supplemental table IV).

When only a prolonged QTc interval was considered, the interval (years) between the ECG- SR and the ECG-AF was 1.16 (2.64 to 0.53) in the group with prolonged QTc (n=185) and 2.58 (6.02 to 0.79) (p<0.0001) in those without a prolonged QTc.

## Discussion

Our main finding is that QTc duration was associated with future incident AF in a cohort of 60-year-olds without known cardiovascular diseases, while no significant association with the risk of incident ischaemic stroke was observed in study participants who were not diagnosed with AF during the follow-up. However, in an independent cohort of patients with AF, we retrospectively revised ECG recorded in sinus rhythm and observed a correlation between the QTc duration and the time elapsed between the registration of the two ECGs, the ECG recorded in sinus rhythm and the ECG at the time of AF diagnosis. Hence, the QTc duration might be considered an AF intermediate phenotype more than an AF predictor. An immediate clinical implication of this observation might be that when serial ECGs are available and a clinical suspicion of paroxysmal AF exists, the simple measure of the QTc duration may strengthen the indication for the AF screening, shorten the time to AF diagnosis and, as a consequence, improve prevention of stroke by initiating anti-coagulant treatment.

Diagnosis of AF in observational studies is often hampered by the paroxysmal nature of the disease and the heterogeneity of the clinical presentation. The LOOP study, a randomised controlled trial, investigated the natural history of silent AF in a population older than 70 years old with an implantable loop recorder during a median follow-up time of 40 months.^16^ In 90% of newly diagnosed patients with AF, symptoms were absent at debut and 87% of patients with AF reported no symptoms at follow-up. We had the unique opportunity to retrospectively analyse the QTc duration in a population where AF was diagnosed by ECG in 100% of the study participants and where the time between two successive ECGs, the first one in SR and the second one when AF was diagnosed, was known. We observed that the QTc duration was inversely correlated with the time interval between the two ECGs: the longest QTc interval the shortest the time between the two ECGs. This indicates that either the QTc duration mirrors an altered cardiac electrophysiology in proximity of the AF diagnosis or that at that time the ECG was recorded, the patient already suffered from a paroxysmal sub-clinical AF. In this perspective, the QTc duration might be a consequence of an already on going cardiac electrical and structural remodelling of the heart leading and contributing to AF/AFL relapses^17^ rather than a potential cause of AF. Consistently, a prolongation of the QTc interval length after conversion of AF to sinus rhythm has been reported.^18 19^

The QTc interval corresponds to the repolarisation of the ventricles and multiple mechanisms may contribute to its prolongation.^20^ Even if the extent to which ventricular and atrial repolarisation share the same ion current is not entirely known,^21^ several risk factors for AF such as age,^22^ hypertension and ischaemic cardiomyopathy^20^ as well as LVH^23^ may associate with a prolonged QTc duration. Consistently, on average the QTc interval was longer in the patients from the Carebbean-e, who are older and had higher prevalence of cardiovascular comorbidities^9^ as compared with the 60YO.

We did not observe an increased risk of ischaemic stroke associated with the QTc duration in the 60YO. In the presence of a concomitant AF diagnosis, we observed a nominally statistical significant association in the 60YO only when QTc duration was introduced in the model as a continuous variable. In Carebbean-e study, QTc duration did not associate with the risk of ischaemic stroke in the presence of AF and no difference in the QTc duration was observed between patients who did and did not suffer from ischaemic stroke during follow-up. Duration of QTc was associated with the risk of ischaemic stroke in both the Multi-Ethnic Study of Atherosclerosis (MESA)^7^ and Reasons for Geographic and Racial Differences in Stroke^8^ studies after adjustment for the presence of AF. Several differences among these study populations may explain the differences observed. The cardiovascular risk profile of the REGARDS is very different from both the risk profile of the 60YO and the Carebbean-e with regard to age, prevalence of cardiovascular risk factors and cardiovascular diseases. In addition, the outcome in the MESA included both ischaemic and haemorrhagic stroke. All together, these results suggest that at present, the QTc duration cannot be safely considered a predictor of ischaemic stroke in patients with AF.

Our study has strengths and limitations. The 60YO had a high participation rate (77%) and a near complete follow-up due to register linkage to obtain the studied outcomes (99.7%). Age was automatically eliminated as a confounding factor because all participants were of the same age. The Carebbean-e is a very well-characterised patient-based study including hospitalised patients at high risk of cardiovascular events because of age and comorbidities. On the other side, this may limit the generalisability of our findings because we only included patients who had at least had one ECG recorded at our hospital before the AF diagnosis and we cannot exclude that they might have had at least an ECG recorded at another hospital or at an outpatient care facility. In the absence of randomisation at inclusion, we cannot rule out if this patient group either had more symptoms related to subclinical AF or was more prone to seek healthcare as compared with the other patients included in the Carebbean-e. However, within the group of patients investigated in this study, this will result in a non-differential bias. An important limitation of our study is that all the ECG-intervals were automatically measured by digital electrocardiographs and not manually confirmed. It is important to tread carefully when looking at the absolute value of the QTc duration reported in this study. We do not propose any cut-off value and focus instead on the relative differences among the groups. In the 60YO, the same ECG machine was used to record the ECG in all study participants, but different ECG machines have been used in the Carebbean-e study. This may affect the inter-individual variability of the measured ECG parameters. Nonetheless, automatically measured ECG intervals have been shown to be reliable^24^ and the QTc duration measured using different methods showed consistent pattern of association with AF^6^ and stroke.^8^ Importantly as they were measured in the same way in all study participants in both cohorts, this would eventually lead to a non-differential misclassification. We cannot exclude that pharmacodynamic interactions with multiple treatment drugs and other concomitant comorbidities not taken into account in our analysis may have affected the QTc duration. In the 60YO, AF/AFL diagnosis was obtained from the NPR while exclusive primary care diagnoses were not available. This may have introduced a bias where primarily hospitalised patients with AF were recorded as an incident AF case. Of note, in a register study between 2006 and 2010, only 12% of all AF-cases in Stockholm were exclusively recorded in primary care.^25^ An additional limitation is the low number of incident ischaemic stroke registered in both studies.

In conclusion, the bulk of our data indicate that the QTc duration is not a risk predictor but a marker of incipient AF or a sign of paroxysmal AF and as such is a possible risk indicator of future ischaemic stroke.

## Data Availability

Data are available upon reasonable request. Data are available upon reasonable request to the corresponding author.
