# Supplementary material for: Is the association of QTc with atrial fibrillation and stroke in cohort studies a matter of time?
Source: Open Heart. 2022 Sep 28;9(2):e002080. doi: 10.1136/openhrt-2022-002080 (PMC9528598; doi:10.1136/openhrt-2022-002080)
Supplement: Supplementary data [file openhrt-2022-002080supp001.pdf]

SUPPLEMENTAL MATERIAL

Is the association of QTc with atrial fibrillation and stroke in cohort studies a matter of time?

Navid Radnahad [1], Hanne Ehrlinder [1], Karin Leander [2], Johan Engdahl [1], Håkan Wallén [1], Bruna Gigante [1,3]

1 Dept of Clinical Science Danderyd University Hospital, Stockholm, Sweden

2 Unit of Cardiovascular and Nutritional Epidemiology, Institute of Environmental Medicine, Karolinska Institutet, Stockholm

3 Division of Cardiovascular Medicine, Dept of Medicine, Karolinska Institutet, Stockholm Sweden

Index

Supplemental Figure I

Supplemental Table I

Supplemental Table II

Supplemental Table III

Supplemental Table IV

Supplemental Figure I. Flowchart of the inclusion and exclusion of participants from the 60YO (Panel A) and Carebbean-e (Panel B)

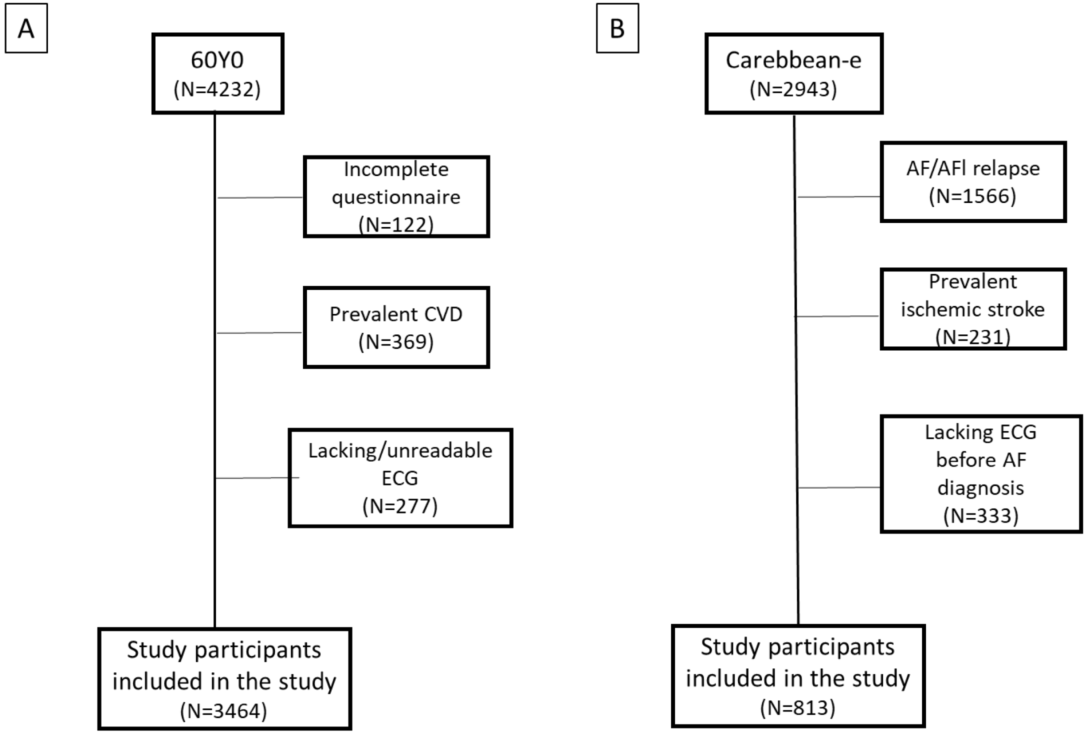

Abbreviations: CVD: cardiovascular disease including myocardial infarction, ischemic stroke and atrial fibrillation/flutter; AF: atrial fibrillation; AFL: atrial flutter; ECG: electrocardiogram

Supplemental Table I. Risk of ischemic stroke, atrial fibrillation and atrial fibrillation and ischemic stroke associated with increasing QTc interval length (ms) in the 60YO.

Atrial Fibrillation	Non Cases/Cases	HR (95%CI)	P value
**Crude**	3029/435	1.01 (1.00-1.01)	<0.0001
**Model 1**	2946/425	1.01 (1.00-1.01)	<0.0001
**Model 2**	2907/421	1.01 (1.00-1.01)	<0.0001
**Ischemic stroke**			
**Crude**	3317/147	1.00 (0.99-1.01)	0.34
**Model 1**	3228/143	1.00 (0.99-1.01)	0.33
**Model 2**	3185/143	1.00 (0.99-1.01)	0.37
**Atrial fibrillation and ischemic stroke**			
**Crude**	3409/55	1.00 (0.99-1.02)	0.06
**Model 1**	3318/53	1.00 (0.99-1.02)	0.06
**Model 2**	3276/52	1.01 (1.00-1.02)	0.04

Model 1: adjusted for sex, hypertension, left ventricular hypertrophy and QRS length.

Model 2: model 1+ diabetes, smoking, BMI, alcohol consumption and hyperlipidemia.

BMJ Publishing Group Limited (BMJ) disclaims all liability and responsibility arising from any reliance placed on this supplemental material which has been supplied by the author(s)

Supplemental Table II. Clinical characteristics of the Carebbean-e study participants included in the present study

	**Carebbean-e (n=813)**
**Age (y)**	83 (79-88)
**Male/Female**	297/516
**BMI (kg/m^2)**	25 (22-28)
**Risk factors, n (%)**	
**Hypertension**	571 (70)
**Diabetes**	119 (15)
**Oral anticoagulant treatment, n (%)**	599 (73)
**ECG parameters**	
**Heart rate, bpm**	71 (62-81)
**PQ, ms**	166 (148-188)
**QRS, ms**	90 (82-100)
**QTc, ms**	433 (414-453)
**Time from ECG in SR to AF diagnosis (y)**	2.16 (5- 0.69)

Continuous data are reported as median and interquartile range. Missing values: BMI, n=7

Supplemental Table III. Association of the QTc duration as a continuous variable and categorized into quartiles with the risk of ischemic stroke in the Carebbean-e study

	**Non cases/ Cases**	**Crude HR (95%CI)**	**Model 1 HR (95%CI)**	**Model 2 HR (95%CI)**
**QTc (ms)**	727/86	1.00 (0.99-1.01)	1.00 (0.99-1.01)	1.00 (0.99-1.01)
**Q1**	181/22	Ref	Ref	Ref
**Q2**	181/20	1.04 (0.57-1.91)	1.00 (0.54-1.84)	1.00 (0.54-1.86)
**Q3**	183/20	1.14 (0.62-2.11)	0.98 (0.52-1.84)	1.01 (0.53-1.93)
**Q4**	182/24	1.43 (0.82-2.57)	1.34 (0.74-2.40)	1.35 (0.69-2.62)

QTc quartiles boundaries (ms): Q1<414; Q2≥414, <433; Q3≥433, <453; Q4≥453

BMJ Publishing Group Limited (BMJ) disclaims all liability and responsibility arising from any reliance placed on this supplemental material which has been supplied by the author(s)

Supplemental Table IV. QTc duration according to time from ECG registration showing sinus rhythm (ECG-SR) to the time when a diagnostic ECG for AF (ECG-AF) was recorded categorized in quartiles (Q) in study participants from the Carebbean-e study

**Time interval between the ECG in sinus rhythm and the AF diagnostic ECG**			**Carebbean-e (n=813)**		**Incident ischemic stroke (n=86)**		**Non cases (n=727)**
**Quartile**	Quartile borders (y)	**N**	**QTc (ms)**	**N**	**QTc (ms)**	**N**	**QTc (ms)**
**Q4**	> 5.07	203	421 (402-436)	17	425 (404-442)	186	420 (402-435)
**Q3**	≤5.07->2.16	203	433 (415-452)	26	432 (424-44)	177	434 (415-452)
**Q2**	<2.16- >0.69	205	440 (420-462)	21	439 (420-460)	184	440 (420-464)
**Q1**	<0.69	202	440 (418-459)	22	441 (412-466)	180	440 (418-457)
